# Myocarditis as a Complication of Campylobacter jejuni-Associated Enterocolitis: A Report of Two Cases

**DOI:** 10.7759/cureus.36171

**Published:** 2023-03-15

**Authors:** Mohamed Zakee Mohamed Jiffry, Nkechi A Okam, Jonathan Vargas, Faith A Adekunle, Stephanie C Pagan, Faisal Khowaja, Mohammad A Ahmed-Khan

**Affiliations:** 1 Internal Medicine, Danbury Hospital, Danbury, USA; 2 School of Medicine, American University of the Caribbean, Cupecoy, SXM; 3 Internal Medicine, University of Vermont, Burlington, USA

**Keywords:** campylobacter extraintestinal manifestations, campylobacter enteritis, campylobacter enteritis myocarditis, enterocolitis, adult cardiology

## Abstract

Myocarditis refers to inflammation of the heart muscle and may occur individually or together with pericarditis, which refers to inflammation of the saclike tissue layer that surrounds the heart. They may have infectious or non-infectious etiologies. *Campylobacter jejuni*, a major cause of gastroenteritis worldwide, may also cause myocarditis in rare situations. We present two cases highlighting this rare complication of diarrheal disease caused by *Campylobacter jejuni* infection and subsequent development of myocarditis. Both patients presented with chest pain and multiple episodes of watery diarrhea, with initial EKGs showing ST segment changes, as well as elevated inflammatory markers and elevated troponins. GI panels for both patients were positive for *Campylobacter jejuni*. Based on their presentations and investigative findings, they were diagnosed with myocarditis secondary to *Campylobacter *infection, and their symptoms subsided with appropriate management. It is unclear if the myocardial damage, in this case, is a direct effect of the toxin on cardiac myocytes or secondary to an immunologic phenomenon. Regardless, *Campylobacter jejuni-*associated myocarditis remains a rare phenomenon and needs to be considered in the differential of patients presenting with concurrent chest pain and diarrheal symptoms.

## Introduction

Myocarditis refers to inflammation of the heart muscle and may occur individually or together with pericarditis, which refers to inflammation of the saclike tissue layer that surrounds the heart [1]. They may be of infectious or non-infectious etiologies. This life-threatening condition can enlarge and weaken the heart muscle limiting the heart’s ability to pump blood sufficiently. 

The most common infectious cause in myocarditis is most often viral and these include agents like Coxsackievirus, Influenza, cytomegalovirus, and HIV, with other uncommon causes being bacterial agents like *Salmonella*, *Shigella*, and *Campylobacter jejuni* [1,2]. Noninfectious causes include cardiotoxins, certain drugs, radiation therapy, chemotherapy, and some systemic disorders [1,2].

Myocarditis is a rare known complication of *Campylobacter jejuni *Infection, and the underlying association is not clearly understood [3]. It is unclear if myocardial damage is caused by a direct effect of the bacteria or toxin upon cardiac myocytes, or if it is secondary to an immunological phenomenon [3]. Patients may present with chest pain, fatigue, shortness of breath, elevated myocardial enzymes, arrhythmia, and/or congestive heart failure, one to two weeks following symptoms of gastroenteritis [4-5]. 

We present two cases highlighting this very rare and potentially life-threatening link between diarrheal disease caused by *Campylobacter jejuni *and myocarditis.

## Case presentation

Case 1

A 35-year-old male with no significant past medical history presented to emergency services with a chief complaint of chest pain. He reported his chest pain started the evening prior to the day of presentation and described it as a non-radiating pressure-like substernal chest pain, which worsened with lying flat and improved on leaning forward. He also reported multiple episodes of watery diarrhea, which started the day prior to presentation and was associated with a fever of 102 F, chills, fatigue, difficulty breathing, generalized body aches, and nausea. He reported eating a whole chicken prepared by his wife earlier that week. He has no family history of premature cardiac disease. He denied any flu-like infection, recent ill contacts, recent travel, or changes in his diet. 

**Figure 1:**
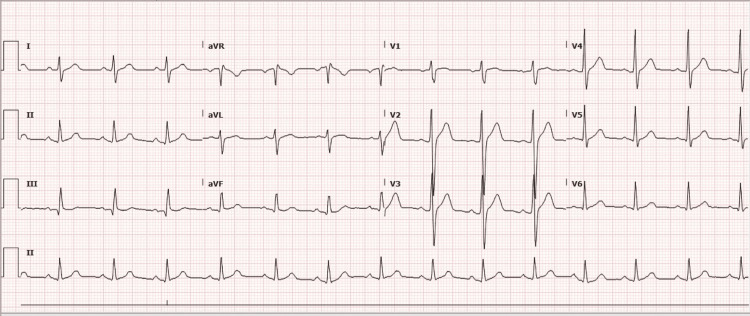
EKG findings of nonspecific ST elevation in leads V2-V5 EKG: Electrocardiogram

Physical examination revealed an alert and interactive male in mild distress. Lungs were clear to auscultation. Cardiovascular exam revealed a regular rate and rhythm with a normal S1 and S2 and no added sounds. No murmurs were appreciated. Pulses were 2+ bilaterally and symmetric. On admission, an electrocardiogram (EKG) showed nonspecific ST segment elevation in leads V2-V5 (Figure 1). 

The laboratory studies were notable for an elevated troponin of 160 ng/L, erythrocyte sedimentation rate (ESR) of 33, C-reactive protein (CRP) of 150, and a predominantly neutrophilic leukocytosis of 14.7. A GI panel was obtained. He was managed conservatively with analgesics, antacids, and fluids. The patient was admitted to the cardiac care unit for further workup. 

On day 2 of admission, troponins trended down to 140 ng/L (normal <22ng/L). Transthoracic echocardiography (TTE) was performed. It showed an ejection fraction of 60-65% with no regional wall motion abnormalities and no pericardial effusion. The GI panel returned positive for *Campylobacter jejuni*. He was initiated on azithromycin 500 mg daily and metronidazole 500 mg twice daily. He was continued on Ibuprofen 800 mg every eight hours and pantoprazole 40 mg daily as part of his management. Continuous telemetry monitoring showed no arrhythmias. Daily monitoring of ESR and CRP was performed, which showed a downtrend. 

Over the next two days, the patient reported resolution of his chest pain and diarrhea. He completed a three-day course of azithromycin while in the hospital. On the fourth day, he was discharged on ibuprofen, pantoprazole, and metronidazole to complete a total 10-day course of antibiotics. He was also scheduled to follow up outpatient with the cardiology service. On a follow-up visit, a repeat EKG showed resolution of the ST segment elevations. ESR and CRP normalized after two weeks of treatment, so Ibuprofen therapy was stopped.

Case 2

A 21-year-old male woke up at 6 AM in the morning with chest pain radiating to his left arm. He had never experienced this type of pain before. He had associated shortness of breath with lightheadedness and heart racing as well. He reported having recently returned from France three days ago, and further stated that while he was vacationing there he remembered having eaten a pizza following which he developed symptoms of gastrointestinal distress and started to have nausea and vomiting with 10-15 episodes of watery diarrhea on a daily basis. His vomitus was non-bloody and nonbilious. Although he did notice fevers two days prior to the presentation, he did not measure them and has not had a fever since.

The patient presented to the emergency department following the onset of chest pain and noted some reduction in the intensity of pain on arrival. He was otherwise well with no significant past medical history. He smoked five cigarettes and a joint of marijuana daily and reported his last use of cocaine was three months ago. He consumed alcoholic beverages one to two times per week. He had no significant family history of sudden cardiac death. No history of allergies was reported. He took no other medications.

**Figure 2:**
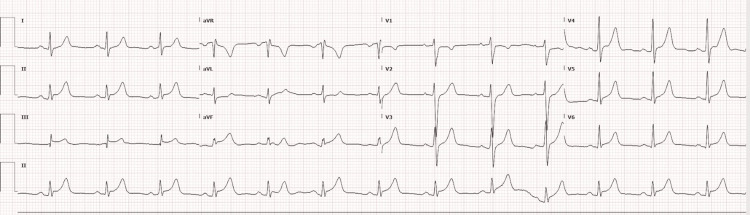
EKG finding of ST segment elevation in leads II, III, and aVF with reciprocal ST depressions noted in lead I and aVL EKG: Electrocardiogram

Examination revealed an alert and interactive young male in mild distress. Respiratory exam did not show evidence of accessory muscle use or respiratory distress. Lung auscultation was clear bilaterally with no wheezes or rales being appreciated. Cardiovascular exam revealed a regular rate and rhythm with a normal S1 and S2 and no added sounds. No murmurs were appreciated. Pulses were 2+ bilaterally and symmetric. An admission ECG revealed ST segment elevation in leads II, III, and aVF with reciprocal ST depressions noted in lead I and aVL (Figure 2).

Bedside echocardiography was done, which showed wall motion abnormalities with likely inferolateral akinesis.

The patient was given loading doses of aspirin and ticagrelor before being emergently taken to the cardiac catheterization laboratory with an operating diagnosis of acute myocardial infarction and catheterization of the left and right coronary circulation was performed, which revealed a normal coronary circulation with no significant coronary artery disease being identified.

Further investigations included high-sensitivity fifth-generation troponin T, which was elevated at 657 ng/L and subsequently up-trended to 818 six hours later, representing a greater than 20% delta. A CRP level was elevated at 59 mg/L (upper limit 8). In view of his GI symptoms, a GI polymerase chain reaction (PCR) panel was sent, which was positive for *Campylobacter jejuni*. Other investigations including complete blood count and chemistry were within normal limits.

Transthoracic echocardiogram revealed borderline normal systolic function with a visually estimated ejection fraction of 50-55%. The left ventricular cavity size was normal with focal areas of severe hypercontractility in the inferior, inferolateral, and lateral walls. The apex and apical segments were also severely hypocontractile. The right ventricular size, wall thickness, systolic function, and systolic pressure were within the normal range. There was no pericardial effusion

In view of these findings, his diagnosis was revised to* Campylobacter* *jejuni-*induced bacterial myocarditis. He was started on low-dose beta-blockade with metoprolol 25 mg twice daily and was subsequently discharged the next day after he remained symptom-free overnight.

## Discussion

*Campylobacter jejuni* is a major cause of gastroenteritis worldwide, transmitted through a contaminated water supply or through ingestion of unpasteurized raw milk and poultry [6]. Myocarditis is a rare but recognized complication of *Campylobacter jejuni *Infection, although the underlying association is not clearly understood [3]. The two prevailing hypotheses put forward so far suggest either an immune-mediated injury to the cardiac tissue (which is also seen in *Campylobacter*-associated reactive arthritis and Guillain-Barre syndrome) or a result of direct bacterial invasion or bacterial toxin invasion of the myocardium [3]. Most immune-mediated responses take time to build up over the course of an infection, and this may further support the hypothesis of an immune-mediated mechanism in those cases of myocarditis associated with *Campylobacter jejuni* enterocolitis that have a delayed presentation weeks after the diarrheal illness. Histopathological analysis of the myocardial tissue in a reported death as a result of myocarditis with *Campylobacter jejuni *enterocolitis revealed marked neutrophilic infiltration and inflammatory changes that were different from those seen in cases of viral myocarditis [3]. Florid acute myocarditis with no detection of *Campylobacter jejuni *on PCR studies indicated the lesser role of direct invasion and the greater role of the toxins from the infective organism [3].

The incidence of myocarditis in confirmed cases of *Campylobacter jejuni*-associated enterocolitis is reportedly low. A Danish study that matched 6204 *Campylobacter jejuni *stool-positive patients against 62,040 control subjects found an incidence rate of 16.1 (95% confidence interval (CI) = 2.3-114.4) per 100,000 person-years in the *Campylobacter jejuni *group compared to 1.6 (95% CI = 0.2-11.4) per 100,000 person-years in the control cohort. Importantly, the study failed to demonstrate a significant difference in the incidence of myocarditis between the two sampled populations [7]. A major limitation of this research, however, was its statistical imprecision due to the rare occurrence of myocarditis in the study population. 

The diagnosis of acute myocarditis is made based on clinical presentation, laboratory values, and cardiac imaging. Patients can present with nonspecific signs and symptoms which may be concerning for acute coronary syndrome. Elevated levels of troponin I are reasonably specific for myocarditis, but they are not sensitive [8], with higher levels being progressively more indicative of myocarditis. White cell count, ESR, and serum CRP are some of the other increased serologic indicators with limited diagnostic value [8]. An EKG could show arrhythmias, AV blocks, and nonspecific repolarization abnormalities but this is not a definitive diagnostic tool as the EKG could also be normal [9]. An echocardiogram may show regional wall motion abnormalities with or without reduced ejection fraction, although left ventricular dysfunction is uncommon and is rare at the time of presentation [10]. Cardiac MRI is mostly used to check for suspected myocarditis as it is a non-invasive tool and can provide significant confirmation of myocardial inflammation, although its sensitivity remains low [3]. Endomyocardial biopsy is the gold standard for diagnosing myocarditis [11]. A biopsy is indicated when severe heart failure is encountered and the benefits of the biopsy outweigh the risks [12]. However, because of the variation in the distribution of inflammation across the myocardium, a histological study of tissue specimens might be ambiguous and it can be considered a reason to avoid biopsy in uncomplicated cases [3].

The majority of cases of *Campylobacter jejuni-*induced myocarditis are usually mild and self-limiting. The mainstay of treatment in healthy individuals is electrolyte replacement and hydration. In higher-risk patients, such as the elderly and immunocompromised, or in those who have more severe symptoms of illness such as fever, bloody stools, or excruciating stomach discomfort, antibiotics are usually considered. *Campylobacter jejuni* infections are best treated with macrolide antibiotics, as resistance to macrolide antibiotics has remained as low as 1-3% in the United States [13]. 

It is important to identify which of the layers of the heart is predominantly inflamed as that may play a role in the initiation of nonsteroidal anti-inflammatory drugs (NSAIDs), such as ibuprofen. In myocarditis, NSAIDs should be used cautiously because in animal models they were shown to enhance the myocarditic process and may increase mortality [14]. Therefore, wherever possible, lower anti-inflammatory doses should be taken into account for perimyocarditis, as its main use is to control symptoms [14]. This is in contrast to acute pericarditis, where NSAIDs should be taken for one to two weeks, usually with a proton pump inhibitor, and then tapered down once inflammation subsides [10]. Current consensus guidelines advise exercise limitation for all patients for the duration of symptoms and for at least three months in athletes [10].

## Conclusions

*Campylobacter jejuni *is a well-known causative agent of gastroenteritis, which is commonly caused by eating contaminated poultry. Myocarditis is a rare complication of *Campylobacter* infection. Both patients in this report were diagnosed with myocarditis induced by *Campylobacter jejuni *based on their clinical history, EKG findings, and laboratory results. Although both patients had similar clinical presentation and laboratory findings, differences were noted with respect to the echocardiogram depicting regional wall motion abnormalities in the latter case, which was managed with beta-blockers in addition to symptomatic treatment. Differentiating between pericarditis and myocarditis is important in the management as NSAIDs are generally avoided in myocarditis whereas they are a mainstay of treatment in pericarditis. 

Although the clinical course and manifestations are generally benign, they could lead to severe heart failure and death. Therefore, it is crucial to have a high index of suspicion and to use resources promptly in order to identify such potentially fatal complications of *Campylobacter jejuni *especially when patients present with chest pain following gastroenteritis.
